# Comparison of metagenomes from fermentation of various agroindustrial residues suggests a common model of community organization

**DOI:** 10.3389/fbioe.2023.1197175

**Published:** 2023-05-10

**Authors:** Kevin S. Myers, Abel T. Ingle, Kevin A. Walters, Nathaniel W. Fortney, Matthew J. Scarborough, Timothy J. Donohue, Daniel R. Noguera

**Affiliations:** ^1^ Great Lakes Bioenergy Research Center, University of Wisconsin-Madison, Madison, WI, United States; ^2^ Wisconsin Energy Institute, University of Wisconsin-Madison, Madison, WI, United States; ^3^ Department of Civil and Environmental Engineering, University of Wisconsin-Madison, Madison, WI, United States; ^4^ Department of Civil and Environmental Engineering, University of Vermont, Burlington, VT, United States; ^5^ Department of Bacteriology, University of Wisconsin-Madison, Madison, WI, United States

**Keywords:** microbiome, fermentation, chain elongation, agroindustrial residue, metagenomics

## Abstract

The liquid residue resulting from various agroindustrial processes is both rich in organic material and an attractive source to produce a variety of chemicals. Using microbial communities to produce chemicals from these liquid residues is an active area of research, but it is unclear how to deploy microbial communities to produce specific products from the different agroindustrial residues. To address this, we fed anaerobic bioreactors one of several agroindustrial residues (carbohydrate-rich lignocellulosic fermentation conversion residue, xylose, dairy manure hydrolysate, ultra-filtered milk permeate, and thin stillage from a starch bioethanol plant) and inoculated them with a microbial community from an acid-phase digester operated at the wastewater treatment plant in Madison, WI, United States. The bioreactors were monitored over a period of months and sampled to assess microbial community composition and extracellular fermentation products. We obtained metagenome assembled genomes (MAGs) from the microbial communities in each bioreactor and performed comparative genomic analyses to identify common microorganisms, as well as any community members that were unique to each reactor. Collectively, we obtained a dataset of 217 non-redundant MAGs from these bioreactors. This metagenome assembled genome dataset was used to evaluate whether a specific microbial ecology model in which medium chain fatty acids (MCFAs) are simultaneously produced from intermediate products (e.g., lactic acid) and carbohydrates could be applicable to all fermentation systems, regardless of the feedstock. MAGs were classified using a multiclass classification machine learning algorithm into three groups, organisms fermenting the carbohydrates to intermediate products, organisms utilizing the intermediate products to produce MCFAs, and organisms producing MCFAs directly from carbohydrates. This analysis revealed common biological functions among the microbial communities in different bioreactors, and although different microorganisms were enriched depending on the agroindustrial residue tested, the results supported the conclusion that the microbial ecology model tested was appropriate to explain the MCFA production potential from all agricultural residues.

## 1 Introduction

Finding ways to generate chemicals and chemical precursors from renewable sources is an important step towards creating a sustainable circular economy that decreases society’s dependance on fossil fuels. Medium chain fatty acids (MCFAs) are one such class of product that can be microbially produced, have applications in lubricant synthesis, production of herbicides and antimicrobials, and can be further processing into additional chemicals (Sarria et al., 2017; Scarborough et al., 2018b). Microbes and microbial communities can produce MCFAs using a wide variety of carbohydrate-rich substrates, making biological MCFA production an attractive target due to the widespread availability of carbohydrate-rich organic wastes that can be used as substrates, such as undistilled corn beer (Ge et al., 2015), thin stillage (Fortney et al., 2021), lignocellulosic fermentation conversion residues (Scarborough et al., 2018a; Scarborough et al., 2018b), a soluble fraction of municipal solid waste (Grootscholten et al., 2013; Grootscholten et al., 2014) and winery residue (Kucek et al., 2016b). In addition to MCFAs, other fermentation products have been identified as coproduced by microbial communities that generate MCFAs from various substrates, including the accumulation of acetic, lactic, succinic, and butyric acids, as well as ethanol (Han et al., 2018; Fortney et al., 2021). Lactic, succinic, and butyric acids can be used as building blocks for materials such as bioplastics (Harmsen et al., 2014). Further, both lactic acid and ethanol have been shown to be intermediate metabolites during MCFA production by members of microbial communities that perform reverse ß-oxidation, also known as chain elongation (Agler et al., 2012; Zhu et al., 2015; Kucek et al., 2016a; Han et al., 2018). Although most MCFA production research has been conducted with microbial communities, it is not clear how to steer a community towards maximizing MCFA production without accumulation of other fermentation products, or how to harness the microbial community to produce primarily one fermentation product. Therefore, additional knowledge is needed to enable the engineering of microbial communities to produce the desired fermentation products. We are interested in generating models that can explain and possibly predict the relationship of microbial community structure with the type of carbohydrate-rich substrates and the type of fermentation products that accumulate.

An emerging microbial ecology model describes three main functions in a chain elongation microbiome; one group of microbes that can ferment carbohydrates to lactic acid but cannot perform chain elongation, other microbes that can perform chain elongation using lactic acid as an electron donor, and others that can perform chain elongation directly from carbohydrates (Scarborough et al., 2018a). This model, initially proposed based on experiments using xylose-rich organic residues from lignocellulosic ethanol production (Scarborough et al., 2018a), has been suggested for other substrates (Crognale et al., 2021; Fortney et al., 2021; Ingle et al., 2021), and there is emerging evidence of MCFA-producing microbes with the genomic capacity for producing MCFA from both lactic acid and carbohydrates (Kang et al., 2022; Wang et al., 2022). In other cases, it is proposed that ethanol can be used as an electron donor and act as an intermediate during MCFA production (Agler et al., 2012; Kucek et al., 2016a). To evaluate whether this microbial ecology model can be generalized to conceptually explain MCFA production from a variety of carbohydrate-rich organic residues, we evaluated the microbial communities that were enriched when the same inoculum was used in bioreactor experiments that fermented several agroindustrial residues, including thin stillage from starch ethanol production (Fortney et al., 2021; Fortney et al., 2022), thin stillage from cellulosic ethanol production (Scarborough et al., 2018a; Scarborough et al., 2020), xylose (Scarborough et al., 2022), dairy manure hydrolysate (Ingle et al., 2021; Ingle et al., 2022), and ultrafiltered milk permeate (Walters et al., 2022; Walters et al., 2023). In all cases, the inoculum was from an acid-phase anaerobic digester at the local wastewater treatment plant (Madison, WI, United States).

Here we present the comparison of metagenome assembled genomes (MAGs) from these bioreactors and examine the role of different microbial groups in the fermentation and chain elongation processes. For this analysis, we developed a script to identify genes encoding key metabolic enzymes in the MAGs and a machine learning algorithm to bin each MAG into relevant categories. This analysis revealed patterns showing that in fermentations in which MCFA is the primary product that accumulates, and the feedstock is a carbohydrate-rich substrate, the microbial ecology model that describes chain elongation occurring via utilization of intermediates or direct utilization of carbohydrates is applicable, even though different microorganisms were enriched depending on the agroindustrial residue tested.

## 2 Materials and methods

### 2.1 Metagenome assembled genome (MAG) sources

MAG data was obtained from previously published lab-scale bioreactor studies of microbial communities grown with various agroindustrial residues (Scarborough et al., 2018a; Scarborough et al., 2020; Fortney et al., 2021; Ingle et al., 2021; Fortney et al., 2022; Ingle et al., 2022; Scarborough et al., 2022; Walters et al., 2022). The operational conditions of the bioreactors are summarized in Table 1 and additional information on sample collection can be found in the respective publications. MAGs were obtained from the inoculum source (two samples, 10 MAGs) (Ingle et al., 2022) and bioreactors fed cellulosic ethanol thin stillage (six samples, 10 MAGs) (Scarborough et al., 2018a; Scarborough et al., 2020), synthetic medium containing xylose as the primary carbon source (three samples, 8 MAGs) (Scarborough et al., 2022), hydrolysate from dairy manure (four samples, 38 MAGs) (Ingle et al., 2021; Ingle et al., 2022), ultra-filtered milk permeate (34 samples, 123 MAGs) (Walters et al., 2022; Walters et al., 2023), and starch ethanol thin stillage (31 samples, 51 MAGs) (Fortney et al., 2021; Fortney et al., 2022). In all cases, only the best-quality representative MAGs determined in each study were used. In total, we used an initial dataset of 240 MAGs from 80 total samples (Table S1).

**TABLE 1:** Bioreactor operational conditions.

Feedstock	Experiment^a^	Main organic substrates in the feedstock	SRT^b^ (days)	HRT^b^ (days)	Temperature	pH	References
Manure Hydrolysate	Manure Hydrolysate	glucose, xylose	6	6	35°C	5.5	Ingle et al. (2021)
Ultra-Filtered Milk Permeate	Milk Permeate 1 (CSTR)	lactose	6	6	35°C	5.5	Walters et al. (2023)
	Milk Permeate 2 (USB)	lactose	>40	0.5	room temp	5.5	This Study
Cellulosic EtOH Thin Stillage	Cellulosic-EtOH Thin Stillage	xylose	6	6	35°C	5.5	Scarborough et al. (2018a), Scarborough et al. (2020)
Xylose Synthetic Medium	Xylose	xylose	6	6	35°C	5.5	This Study
Starch EtOH Thin Stillage	Starch-EtOH 1	glycerol, carbohydrates, lactic acid	6	6	35°C	5.5	Fortney et al. (2021)
	SR-Starch-EtOH 2	glycerol, carbohydrates, lactic acid	6	6	35°C	5.5	Fortney et al. (2021)
	SR-Starch-EtOH 3	glycerol, carbohydrates, lactic acid	1	1	35°C	5.5	Fortney et al. (2021)
	SR-Starch-EtOH 4	glycerol, carbohydrates, lactic acid	6	6	55°C	5.0	Fortney et al. (2021)
	SR-Starch-EtOH 5	glycerol, carbohydrates, lactic acid	1	1	55°C	5.0	Fortney et al. (2021)

^a^ CSTR, continuously stirred tank reactor; USB, upflow sludge blanket reactor; SR, solids removed from the thin stillage by decanting.

^b^ SRT, solid retention time; HRT, hydraulic retention time.

### 2.2 MAG dereplication and taxonomic classification

The program dRep (v3.2.2; *dereplicate* command) (Olm et al., 2017) was used to identify redundant MAGs using default settings, except *-conW* was set to 0.5 and *-N50W* was set to 5. This reduced the total MAG number from 240 to 217 non-redundant MAGs (Supplementary Table S2). CheckM (v1.0.11; *lineage_wf* and *qa* commands with default parameters) (Parks et al., 2015) was used to determine relevant quality parameters for each of the 217 MAGs (Supplementary Table S2). All 217 MAGs were taxonomically classified using GTDB-Tk (v1.5.1; database release 202; *classify_wf* command with default parameters) (Supplementary Table S3).

### 2.3 Alignment and relative abundance calculations

To predict the relative abundance of microorganisms represented by the 217-MAG dataset in samples from the different bioreactors, the genome FASTA files of all the MAGs were concatenated, and then Bowtie2 (v2.2.2 with default parameters) (Langmead and Salzberg, 2012) was used to align the FASTQ sequencing files. Resulting SAM files were converted into BAM files and sorted using samtools (v1.15.1; *view* and *sort* commands with default parameters) (Li et al., 2009). CoverM (v0.4.0; *coverm genome* command with default parameters) (https://github.com/wwood/CoverM) was used to generate relative abundance statistics of mapped reads in the sorted BAM files (Supplementary Table S2). We identified 131 MAGs with at least 1% relative abundance in at least one sample across all experiments, which we define as the high-abundance MAG dataset (Supplementary Table S4). A relative abundance of 1% has been used previously as an abundance threshold (Fitzgerald et al., 2015; Scarborough et al., 2018a; Scarborough et al., 2018b; Scarborough et al., 2020).

### 2.4 Phylogenetic analyses

Maximum likelihood phylogenetic trees were generated using RAxML-NG (v0.9.0; model LG + G8+F) (Kozlov et al., 2019) using 1,000 bootstraps. GTDB-Tk (v1.5.1; database release 202; *ani_rep* command with default parameters) (Chaumeil et al., 2019) was used to identify closest related genomes, which were downloaded from NCBI. The MAGs and closest genomes were compared using GTDB-Tk (*identify* and *align* commands with default parameters) using a set of 120 bacterial single-copy marker genes (Bac120) for all trees. *Prevotella intermedia* (GCF_001953955.1) was used as an outgroup to root the trees.

An additional analysis was performed to compare homologs of subunit B of the electron transfer flavoprotein (EtfB). For this, EtfB homologs were identified using known protein sequences (Walters et al., 2023) and tBLASTn (v2.8.1, default parameters) (Camacho et al., 2009) with “pident” (percent identity to the query sequence) > 25% and “qcovhsp” (coverage of the query sequence) > 70%. EtfB homologs were aligned using MUSCLE (v3.8.31, default parameters) and a phylogenetic tree was constructed using RAxML-NG using 500 boostraps. All files used in this analysis are available on GitHub (https://github.com/GLBRC/agroindustrial_residue_metagenomics).

### 2.5 Non-metric multidimensional scaling plots

Non-metric multidimensional scaling (NMDS) plots were generated from the relative abundance calculations for the 217 non-redundant MAGs using R (v4.1.0) (Core Team, 2018). Specifically, the *vegdist* command with the “*bray*” index (from the vegan package, v2.6-4) was used to determine the distance metrics and the *metaMDS* command (from the vegan package, v2.6-4) was used to generate the NMDS values. Plots were constructed using ggplot2 (Wickham, 2016) from the NMDS values and edited for clarity using Adobe Illustrator (v27.2). Statistical comparisons were performed using permutation-based multivariate analysis of variance (PerMANOVA) via the *adonis* command (from the vegan package, v2.6-4) with “*euclidean”* distance and the Benjamini-Hochberg adjustment (adjusted *p*-value <0.05 accepted as significant) (Benjamini and Hochberg, 1995; Anderson, 2017). The R script used to generate the NMDS plot is available on GitHub (GitHub page: https://github.com/GLBRC/agroindustrial_residue_metagenomics).

### 2.6 Homology-based gene identification

A homology-based analysis was performed to identify genes encoding enzymes of fermentation and central carbon metabolism in each MAG. The query protein sequences used were manually vetted through either EcoCyc (Keseler et al., 2011), MetaCyc (Caspi et al., 2020), SWISS-PROT via UniProtKB (Boutet et al., 2016), or other published datasets. Query protein amino acid sequences and metadata were downloaded from the UniProtKB database. tBLASTn (v2.8.1) (Camacho et al., 2009) was used to identify homologs using default parameters. Subject sequences that had an e-value less than 1 × 10^−10^, a “pident” (percent identity to the query sequence) value greater than 25%, and a “qcovhsp” (coverage of the query sequence) value greater than 70% were used to determine gene homologs (Supplementary Table S5). All files and scripts are available on GitHub (GitHub page: https://github.com/GLBRC/agroindustrial_residue_metagenomics).

### 2.7 Multiclass classification machine learning algorithm

MAGs were classified into four functional groups. The first group, “Ferment to Intermediates”, consists of MAGs that ferment carbohydrates into intermediate extracellular products, such as ethanol or lactic acid. The second group, “Intermediate Chain Elongators”, consists of MAGs that convert intermediate extracellular products (e.g., ethanol or lactic acid) into medium chain fatty acids (MCFAs) using reverse ß-oxidation. The third group, “Carbohydrate Chain Elongators”, consists of MAGs that ferment carbohydrates directly into MCFAs. A fourth group, “uninvolved”, was used to bin MAGs that could not be classified into the three functional groups.

Multiclass classification machine learning was utilized to categorize the MAGs based on gene homologs of key fermentation pathways that were detected. A training set was constructed using organisms known to fit into one of the four groups (Supplementary Table S6). *Bifidobacterium* species and lactic acid bacteria were used for the Ferment to Intermediates training set (Okada et al., 1979; Pokusaeva et al., 2011; Pruckler et al., 2015; Tanner et al., 2016; Eckel and Vogel, 2020; Ferrero et al., 2021; Kasmaei et al., 2022; Ksiezarek et al., 2022), *Clostridium* and *Megasphaera* species were used for the Intermediate Chain Elongators training set (Wallace et al., 2003; Seedorf et al., 2008; Jeon et al., 2017; Kobayashi et al., 2017; Tao et al., 2017; Yang et al., 2018; Yoshikawa et al., 2018; Litty and Muller, 2021), *Caproicibacter* and *Roseburia* species were used for the Carbohydrate Chain Elongators training set (Kim et al., 2015; Tamanai-Shacoori et al., 2017; Flaiz et al., 2020; Schoelmerich et al., 2020), and *Acetobacter, Prevotella,* and *Sphaerochaeta* species were used for the uninvolved training set.

Multiple multiclass classification machine learning algorithms were tested using the auto_ml module (v2.9.10) (https://github.com/ClimbsRocks/auto_ml). The algorithms tested against baseline were *Decision Tree* (Pedregosa et al., 2011), *Random Forest* (Pedregosa et al., 2011), *Linear Regression* (Pedregosa et al., 2011), *XGBoost* (https://xgboost.readthedocs.io/en/stable/index.html), *Neural Network* (Pedregosa et al., 2011), *Nearest Neighbors* (Pedregosa et al., 2011), *Extra Trees* (Pedregosa et al., 2011), *CatBoost* (Prokhorenkova et al., 2018), and *LightGBM* (Zhang et al., 2017). The machine learning algorithms were evaluated for correct classification of training set genomes into functional groups using multiple analyses: the logloss metric (-log(*p*), where *p* is the probability of correctly categorizing the training set) (Bian and Tao, 2011) for each algorithm compared to the baseline value of no algorithm, precision-recall (PR) curves for each algorithm and receiver operating characteristic (ROC) curves for each algorithm (Haibo and Garcia, 2009). These evaluations showed that using the *LightGBM* model provided the largest decrease in logloss metric (a 99.91% improvement compared to baseline alone) while maximizing true positives and minimizing false positives. The script, files used for the machine learning analysis, and the results of the multiclass classification machine learning analysis are available on GitHub (GitHub page: https://github.com/GLBRC/agroindustrial_residue_metagenomics).

### 2.9 Hierarchical clustering

MAGs were classified into predicted functional groups using hierarchical clustering based on the detected genes in metabolic pathways important in MCFA production (Walters et al., 2023). Hierarchical clustering was performed in R (v4.1.0) (Core Team, 2018) using the gplots R package (v3.1.3, heatmap.2 command with default parameters, https://github.com/talgalili/gplots/). MAGs were classified using the hierarchical clustering results in the Ferment to Intermediates group if they had high percentage of genes detected in the bifid shunt or phosphoketolase pathways and low percentage of genes detected in the lactic acid utilization and reverse ß-oxidation pathways, in the Intermediate Chain Elongators group if they had low percentage of genes detected in the bifid shunt or phosphoketolase pathways and high percentage of genes detected in the lactic acid utilization and reverse ß-oxidation pathways, and in the Carbohydrate Chain Elongators group if they had low percentage of genes detected in the bifid shunt, phosphoketolase, and lactic acid utilization pathways but high percentage of genes detected in the reverse ß-oxidation pathway (Supplementary Table S2). The script, files used, and results of this analysis are available on GitHub (GitHub page: https://github.com/GLBRC/agroindustrial_residue_metagenomics).

## 3 Results

### 3.1 Analysis of the non-redundant MAG dataset

**FIGURE 1:**
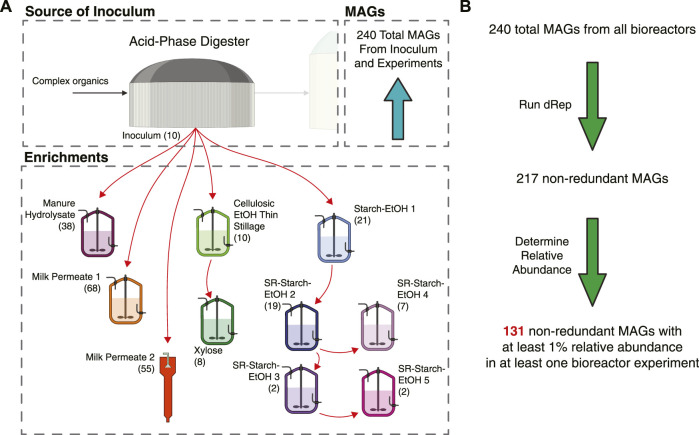
Overview of bioreactors operated with the different agroindustrial feedstocks and their contribution to the non-redundant MAG dataset. **(A)** Graphical overview of inoculum source and enrichments with different feedstocks, indicating the number of MAGs assembled from each source. All reactors were completely mixed flow-through reactors, except for Milk Permeate 2, which was an upflow sludge blanket reactor. See Table 1 for operational conditions. **(B)** Flow chart indicating how the MAGs were filtered for this work. From a total of 240 MAGs, dRep (Olm et al., 2017) was used to identify redundant MAGs and define a set of 217 non-redundant MAGs. Abundance was then used to define a set of 131 high-abundance and non-redundant MAGs.

**FIGURE 2:**
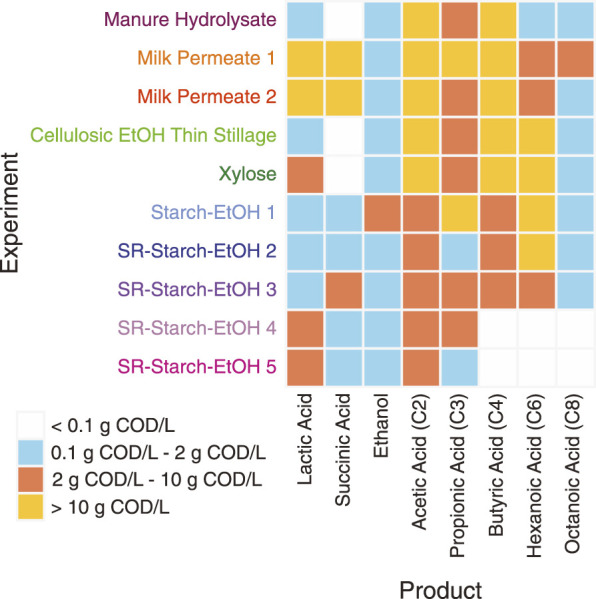
Summary of extracellular fermentation products that accumulated in the bioreactors. Product concentrations, measured in chemical oxygen demand (COD) per liter, are summarized into four groups, indicating maximum concentrations measured during the course of the experiments: <0.1 g COD/L (light grey), between 0.1 and 2 g COD/L (blue), between 2 g COD/L and 10 g COD/L (red), >10 g COD/L (yellow).

For this study we used MAGs assembled from 10 different bioreactors that were fed various agroindustrial residues (Figure 1). The microbial communities that were enriched in these bioreactors originated from the same inoculum source, an acid-phase anaerobic digester used in the solids handling treatment train at the local wastewater treatment plant (Madison, WI, United States). In addition to the type of agroindustrial residue used as the feedstock, parameters such as temperature and pH were also different in some bioreactor experiments (Table 1). Bioreactor performance has been described elsewhere for a bioreactor fed xylose-rich thin stillage from cellulosic ethanol production (Scarborough et al., 2018b), one fed a carbohydrate-rich hydrolysate created from chemical pretreatment of dairy manure (Ingle et al., 2021), five bioreactors fed thin stillage from starch ethanol biorefining (Fortney et al., 2021), and one bioreactor fed lactose-rich ultra-filtered milk permeate (Walters et al., 2023). Two additional bioreactors complete the set of 10 bioreactors used in this study; one fed a xylose-rich synthetic medium and a second one operated with ultra-filtered milk permeate as the feedstock. The MAGs assembled from all of the bioreactors have been reported and are publicly available (Scarborough et al., 2018a; Fortney et al., 2022; Ingle et al., 2022; Scarborough et al., 2022; Walters et al., 2022). The main fermentation products that accumulated in the medium of these bioreactors include lactic and succinic acids, ethanol, as well as the short chain fatty acids (SCFAs) acetic and propionic acids and the MCFAs butyric, hexanoic, and octanoic acids (Figure 2).

Combined, there are a total of 240 MAGs across these bioreactors (Figure 1B; Supplementary Table S1). Given the similarities in the inoculum source and in the accumulated fermentation products, we hypothesized that the MAGs assembled from these microbial communities would have a high degree of similarity. However, when the program dRep (Olm et al., 2017) was used to identify MAGs with at least 99% average nucleotide identity (ANI), only 23 MAGs were highly similar among the 240 MAGs (Figure 1B; Table S1). This dereplication analysis resulted in a library of 217 non-redundant MAGs that we used to further evaluate the microbial communities in the bioreactors (Supplementary Table S2).

This collection of 217 non-redundant MAGs represented median relative abundances ranging from 63.5% to 90.3% in the bioreactor samples, but a median relative abundance of only 11.6% for the inoculum (Table 2). The low percentage for the inoculum indicates that most of the 217 MAGs in the library represented microbial community members that were not abundant in the acid-phase digester inoculum, but were instead enriched during the operation of the bioreactors.

**TABLE 2:** Relative abundance of all 217 non-redundant MAGs across all experiments.

Experiment	Number of MAGs detected as present^a^	Min-max relative abundance range^b^ (%)	Median relative abundance (%)
Inoculum	21	10.3–13.0	11.6
Manure Hydrolysate	99	68.9–77.9	74.7
Milk Permeate 1	148	9.3–91.1	74.6
Milk Permeate 2	139	7.9–80.1	69.2
Cellulosic EtOH Thin Stillage	75	33.0–87.3	86.6
Xylose	21	88.0–88.5	88.5
Starch-EtOH 1	100	8.5–87.0	63.5
SR-Starch-EtOH 2	55	87.9–92.6	90.3
SR-Starch-EtOH 3	52	80.8–88.8	85.2
SR-Starch-EtOH 4	53	84.6–89.7	86.1
SR-Starch-EtOH 5	24	74.9—77.4	76.4

^**a**^ A MAG was defined to be present in a sample if the relative abundance was greater than 0%.

^b^ Minimum and maximum relative abundances represented by the non-redundant MAG dataset among all the samples from each bioreactor experiment and from the inoculum samples.

**FIGURE 3:**
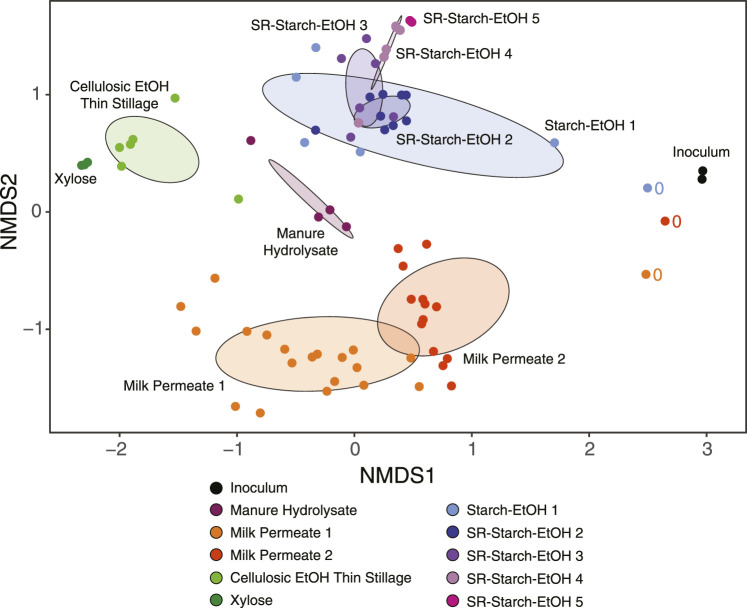
Non-metric multidimensional scaling (NMDS) plot of the relative abundances of the microbial communities using the 217 non-redundant MAGs across all experiments over all measured time points (stress value 0.17). Samples from different bioreactor experiments are color coded according to the key. Ovals represent the standard deviation of the average value for all samples from each bioreactor experiment and are color coded according to the key. Samples that were taken at the time of inoculation are marked with ‘0’. See Table 1 for description of bioreactor operational conditions and definition of experiment names.

A non-metric multidimensional scaling (NMDS) analysis of the relative abundance of MAGs in the analyzed samples reveals divergence in the microorganisms that were enriched during growth in the different agroindustrial residues (Figure 3). The lack of overlap of the abundant MAGs among agroindustrial residue media used indicates that the media played a large role in shaping the microbial communities in these bioreactors. The dataset includes samples collected from bioreactors operated with the same agroindustrial residue but different operational conditions. In these cases, the NDMS plot suggests that agroindustrial residue used had a larger impact in shaping the microbial community compared to the operational condition. For example, several bioreactors were operated using starch ethanol thin stillage (Fortney et al., 2021), and in the NDMS plot (Figure 3) the samples from these bioreactors clustered together and separate from the samples from bioreactors that used other agroindustrial residues (adjusted *p*-value <0.05). The dataset also includes samples collected from bioreactors operated under identical conditions but receiving different agroindustrial residues. This is the case for the Milk Permeate 1, Xylose, and the Starch-EtOH 1 experiments (Figure 3). Although they were all operated under identical conditions, there is no overlap of the abundant MAGs from these reactors in the NDMS plot (adjusted *p*-value <0.05), supporting the argument that the agroindustrial residue used had a larger impact in the microbial communities than the operational conditions used.

The set of non-redundant MAGs has a diverse composition (Table 3; Supplementary Table S3), with MAGs belonging to eight phyla and 12 families within these phyla. 24 MAGs were classified to the genus level based on the coverage in the metagenomic data sets. In addition, this non-redundant set includes MAGs assembled with short-read Illumina (149 MAGs) and long-read PacBio technologies (68 MAGs). Estimates of completion and contamination in this dataset are greater than 75% and less than 7.5%, respectively. The MAGs resulting from Illumina sequencing had assemblies with 1–558 contigs, whereas the MAGs obtained from PacBio sequencing were assembled in 1–44 contigs (Table 3; Supplementary Table S2).

**TABLE 3:** General information on the 217 MAGs.

Characteristic	Value
Phyla Identified	8
Families Identified	12
Genera Identified	24
Illumina Total (contig range)	149 (1-558)
PacBio Total (contig range)	68 (1-44)
Completion Minimum	75%
Contamination Maximum	7.5%

### 3.2 Enzymes in metabolic pathways identified in the non-redundant MAG dataset

**FIGURE 4:**
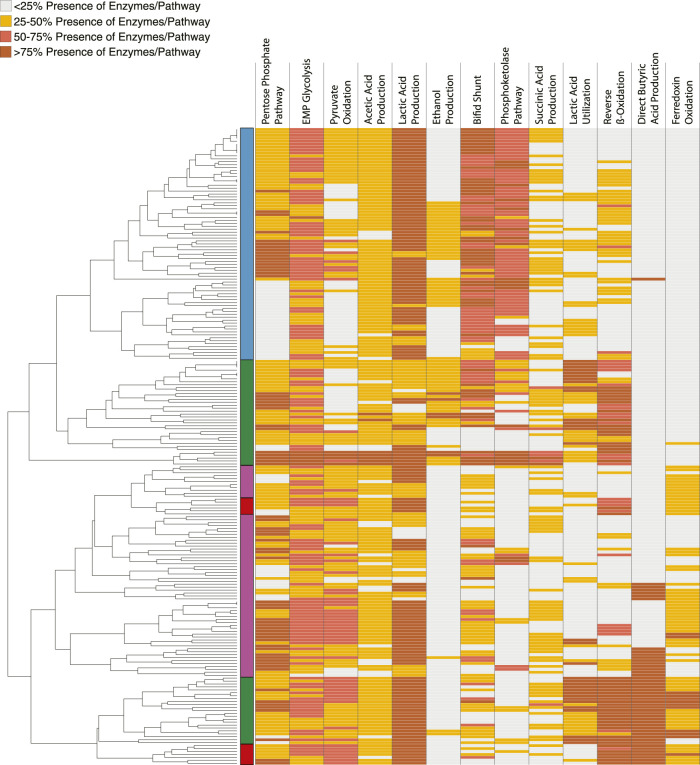
Clustering 217 MAGs using metabolic pathways. Identified homologous proteins in the indicated metabolic pathways (columns) for each of the 217 non-redundant MAGs (rows). Colors represent the percentage of protein homologs for each pathway for each MAG as indicated in the key. The MAGs were hierarchically clustered resulting the dendrogram on the left. Functional group assignments based on hierarchical clustering is indicated on the left, and color coded as Ferment to Intermediates (blue), Intermediate Chain Elongators (green), Carbohydrate Chain Elongators (red), and uninvolved in MCFA production (purple).

We sought to make predictions on the role of different members of the microbial communities enriched in the bioreactors and to evaluate the microbial ecology model for MCFA production that hypothesizes the presence of some community members that produce MCFA directly from carbohydrates (Carbohydrate Chain Elongators), other community members that produce MCFA from lactic acid or ethanol as intermediate fermentation products (Intermediate Chain Elongators), and other community members that produce these intermediate products but do not perform chain elongation (Ferment to Intermediates) (Scarborough et al., 2018a). To this end, we queried the MAGs for the presence of homologs of individual proteins present in different fermentation pathways (Figure 4; Supplementary Table S5) (Walters et al., 2023). This allowed categorization of MAGs by association of similar patterns of the presence of homologous proteins from each metabolic pathway examined. Using the hierarchical clustering of the MAGs based on the percentage of homologs present per pathway, we categorized the MAGs into the functional groups. Based on this analysis, 79 MAGs are predicted to ferment carbohydrates to intermediate products (Ferment to Intermediates), 59 MAGs are predicted to produce MCFA from the intermediate products (Intermediate Chain Elongators), and 13 MAGs are predicted to produce MCFA from carbohydrates (Carbohydrate Chain Elongators, Figure 4; Supplementary Table S2).

### 3.3 Machine learning-based classification

We also wanted to test if we could use multiclass classification machine learning to generate similar predictions, as a way to evaluate large MAG datasets quickly and to remove any bias in functional assignments based on enzyme assignments. For this evaluation, we constructed a training set of isolated organisms predicted to perform the three specific functions in the model, plus organisms not known or likely to participate in these activities (Supplementary Table S6). As input to the machine learning algorithm, we used the information gathered about detection of protein homologs in the metabolic pathways relevant to the ecological model (Supplementary Table S5). The training set was then used to investigate a number of possible multiclass classification machine learning algorithms, with the *LightGBM* algorithm (Zhang et al., 2017) producing the best results of binning the genomes into the correct functional groups based on multiple methods of evaluation (logloss comparison to baseline, PR curve, and ROC curve).

**FIGURE 5:**
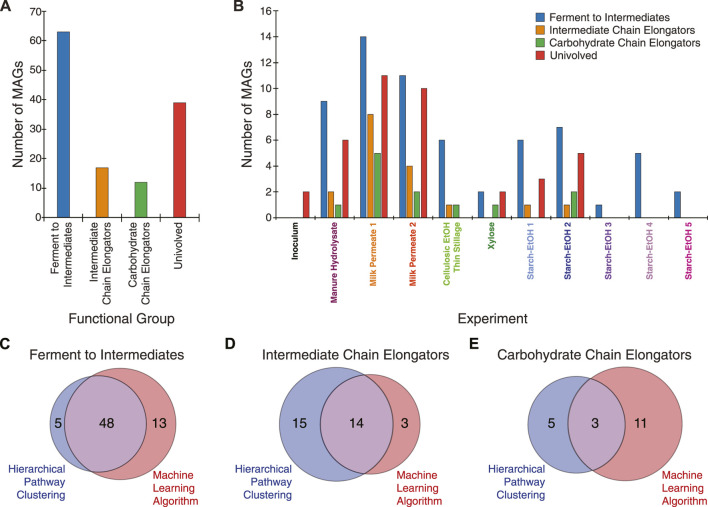
Summary of machine learning categorization. **(A)** Distribution of the 131 MAGs in the different functional groups used for machine learning classification. **(B)** Distribution of the 131 MAGs according to the experiment from which each was identified in according to how they were classified in by machine learning. Venn diagrams show comparison of the hierarchical pathway clustering classification and the machine learning classification for Ferment to Intermediates group **(C)**, the Intermediate Chain Elongators group **(D)**, and the Carbohydrate Chain Elongators group **(E)**. Only MAGs present with at least 1% relative abundance in at least one sample across all experiments are included in the comparison analysis.

To evaluate the machine learning multiclass classifications, a subset of the most abundant MAGs was selected for further analysis. The 217 non-redundant MAGs across the experiments were filtered to include only MAGs with at least 1% relative abundance in at least one experiment sample (Figure 1B; Supplementary Table S4). The resultant 131 high-abundance MAGs include ones assembled from short read Illumina technology (74 MAGs) and long read PacBio technology (57 MAGs) and were categorized into one of four functional groups using the trained multiclass machine learning model. Overall, 63 MAGs were predicted as being able to ferment carbohydrates to intermediate products (Ferment to Intermediates), 17 MAGs were predicted as being able to convert intermediate products to MCFAs (Intermediate Chain Elongators), 12 MAGs were categorized as being able to ferment carbohydrates to MCFAs (Carbohydrate Chain Elongators), and 39 MAGs were predicted not to be involved in MCFA production (Figure 5A; Supplementary Table S4). The MAGs in each category were derived from several different agroindustrial residue experiments (Figure 5B), showing that similar functions occurred with the different agroindustrial residues.

Comparison of the MAGs classified into the functional groups by the machine learning algorithm to classification by hierarchical pathway clustering reveals differences based on the approaches (Figures 5C–E). The Ferment to Intermediates group shows a large amount of overlap between the two methods (Figure 5C). The hierarchical pathway clustering method identified more MAGs than the machine learning algorithm for the Intermediate Chain Elongators group while there was little overlap among the methods for the Carbohydrate Chain Elongators group (Figures 5D, E).

**FIGURE 6:**
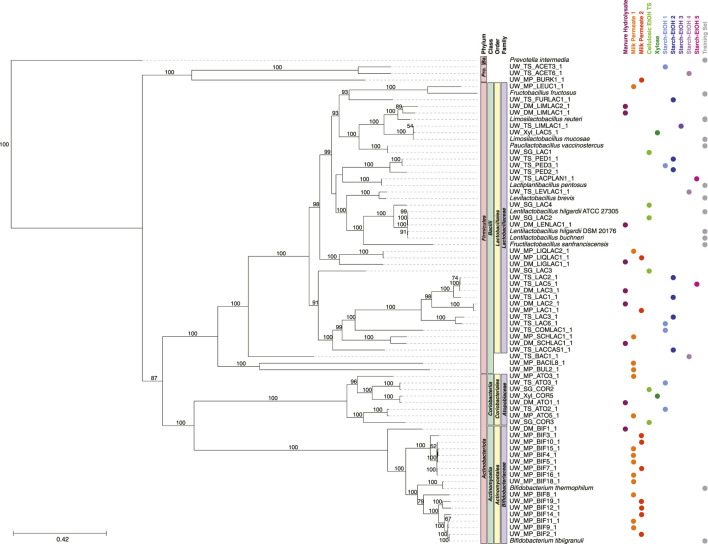
Phylogenetic tree of MAGs classified in the Ferment to Intermediates group and the genomes used in the training set. A maximum-likelihood phylogenetic tree constructed using RAxML-NG (Kozlov et al., 2019) with 1,000 bootstraps (values >50 shown) and using the 120 bacterial housekeeping gene concatenations generated by GTDB-Tk (Chaumeil et al., 2019). Taxonomic classification performed using GTDB-Tk (database version 202) (Chaumeil et al., 2019). The scale bar indicates the number of nucleotide substitutions per sequence site. Genomes used in the training set are shown (labeled Training Set) and NCBI Accession Numbers are found in Supplementary Table S6. Color dots indicate experiment the MAG was identified in (experiments with no MAGs present in the tree are not shown). *Ba.*, *Bacteroidota*; *Pro., Proteobacteria.*

**FIGURE 7:**
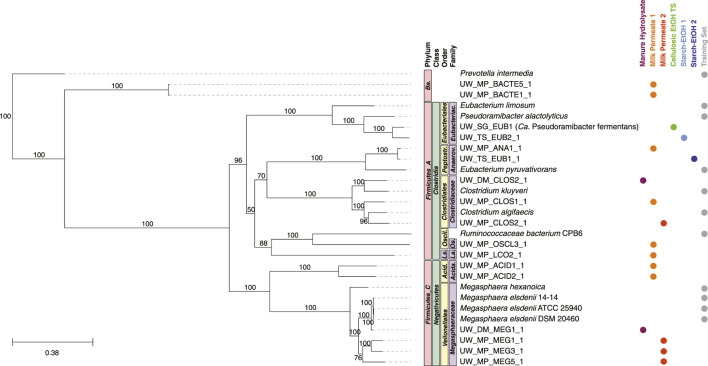
Phylogenetic tree of MAGs classified in the Intermediate Chain Elongators group and genomes used in the training set. A maximum-likelihood phylogenetic tree constructed using RAxML-NG (Kozlov et al., 2019) with 1,000 bootstraps (values >50 shown) and using the 120 bacterial housekeeping gene concatenations generated by GTDB-Tk (Chaumeil et al., 2019). Taxonomic classification performed using GTDB-Tk (database version 202) (Chaumeil et al., 2019). The scale bar indicates the number of nucleotide substitutions per sequence site. Genomes used in the training set for this group are shown (labeled Training Set) and NCBI Accession Numbers are found in Supplementary Table S6. Color dots indicate experiment the MAG was identified in (experiments with no MAGs present in the tree are not shown). *Ba.*, *Bacteroidota*; *Acida., Acidaminococcales; Acida., Acidaminococcaceae; Peptostr., Peptostreptococcales; Anaerov., Anaerovoracaceae; Eubacteriac., Eubacteriaceae; Ls., Lachnospirales; La., Lachnospiraceae; Oscil., Oscillospirales; Os. Oscillospiraceae*.

**FIGURE 8:**
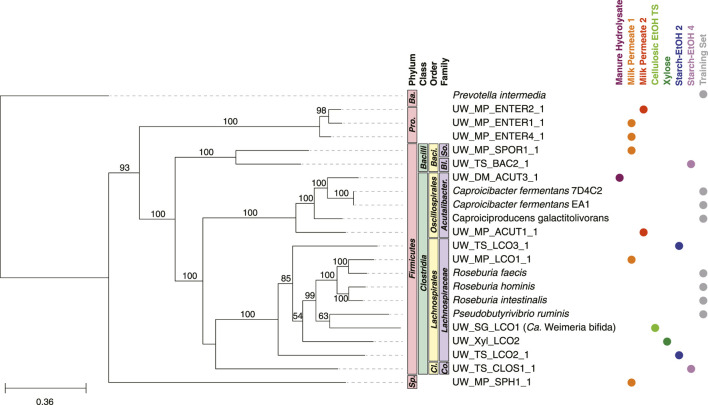
Phylogenetic tree of MAGs classified in the Carbohydrate Chain Elongators group and genomes used in the training set. A maximum-likelihood phylogenetic tree constructed using RAxML-NG (Kozlov et al., 2019) with 1,000 bootstraps (values >50 shown) and using the 120 bacterial housekeeping gene concatenations generated by GTDB-Tk (Chaumeil et al., 2019). Taxonomic classification performed using GTDB-Tk (database version 202) (Chaumeil et al., 2019). The scale bar indicates the number of nucleotide substitutions per sequence site. Genomes used in the training set for this group are shown (labeled Training Set) and NCBI Accession Numbers are found in Supplementary Table S6. Color dots indicate experiment the MAG was identified in (experiments with no MAGs present in the tree are not shown). *Ba.*, *Bacteroidota*; *Pro., Proteobacteria; Baci., Bacillales; So., Sporolactobacillaceae; Bl., Bacillaceae; Acutalibacter., Acutalibacteraceae; Cl., Clostridiales; Co., Clostridiaceae; Sp., Spirochaetota.*

Focusing on the machine learning classification, and to further investigate the MAGs present in functional groups responsible MCFA production, phylogenetic trees were constructed comparing the genomes used in the training set and the MAGs classified into each functional group (Figures 6–8). The MAGs were taxonomically classified using GTDB-Tk (Chaumeil et al., 2019). For each functional group examined, we found multiple taxonomic groups across taxonomic levels, ranging from phyla to family (Figures 6–8). Indeed, a subset of the MAGs in groups share no overlap at the class or family level with genomes in the training set, suggesting the machine learning algorithm is identifying new taxonomic groups that may perform the specific biological function.

### 3.4 MAGs predicted to participate in fermentation to intermediate products

The majority of the MAGs predicted in the Ferment to Intermediates group belonged to the *Lactobacillaceae*, *Bifidobacteriaceae*, and *Atopobiaceae* families (Figure 6). In general, the MAGs in *Bifidobacteriaceae* and *Lactobacillaceae* clustered with the genomes from the same taxonomic group used in the training set. Further, the machine learning algorithm classified MAGs of the *Atopobiaceae* family into this group, despite no member of this family being present in the training set. A small subset of the MAGs in this functional group belonged to other taxonomic groups: class *Bacilli* (3 MAGs) and phylum *Proteobacteria* (3 MAGs) (Figure 6).

### 3.5 MAGs predicted to participate in chain elongation from intermediate products

The majority of the MAGs in the Intermediate Chain Elongators group, predicted to convert fermentation intermediates into MCFAs, were predicted to belong to five families: *Megasphaeraceae, Acidaminococcaceae*, *Clostridiaceae*, *Anaerovoracaceae*, and *Eubacteriaceae* (Figure 7). This included a MAG (UW_SG_EUB1, *Ca.* Pseudoramibacter fermentans) that was studied at the transcriptomic level and predicted to ferment intermediates into MCFAs (Scarborough et al., 2020). The MAGs in four of the five families were clustered with genomes in the same families used in the training set. However, there were no genomes in the training set that belonged to the family *Acidaminococcaceae, Lachnospiraceae*, or *Oscillospiraceae*. Two MAGs belonged to phylum *Bacteroidota* (order *Bacteroidales*).

### 3.6 MAGs predicted to participate in chain elongation from carbohydrates

The MAGs predicted to belong to the Carbohydrate Chain Elongators group, ones which convert carbohydrates directly to MCFAs, belonged primarily to two families: *Lachnospiraceae* and *Acutalibacteraceae* (Figure 8). Included in this group is a MAG (UW_SG_LCO1, *Ca.* Weimeria bifida) previously studied in-depth and suggested to perform chain elongation from carbohydrate substrates (Scarborough et al., 2020). Seven of the MAGs present in the Carbohydrate Chain Elongators group belonged to other taxonomic groups: class *Bacilli*, class *Clostridia* as well as phyla *Proteobacteria* and *Spirochaetota* (Figure 8).

## 4 Discussion

**FIGURE 9:**
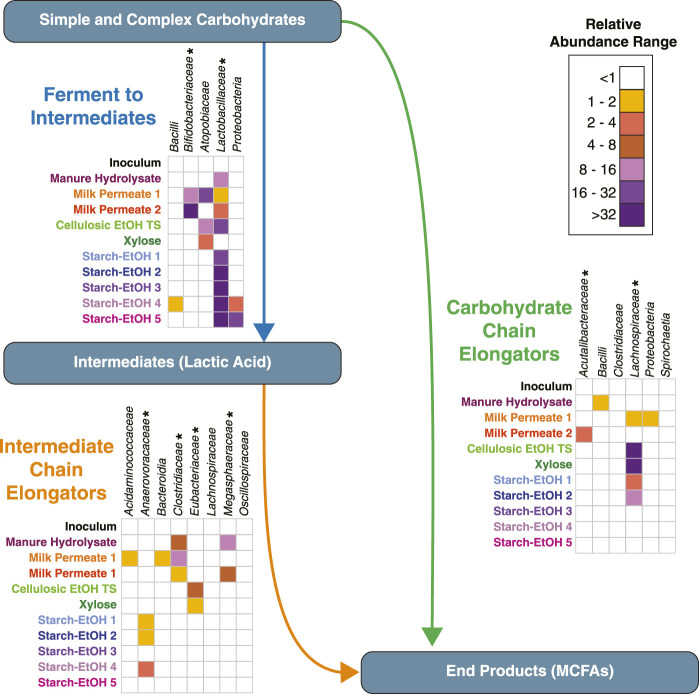
Cumulative relative abundances for taxa within each group reveal common biological functions across agroindustrial residues. Cumulative relative abundances for each taxa across the 10 experiments for MAGs classified in the Ferment to Intermediates group, the Intermediate Chain Elongators group, and the Carbohydrate Chain Elongators group as labeled. For each panel, the heat map represents the cumulative relative abundance, with white indicating a cumulative relative abundance <1. Asterisks indicate taxa present in the machine learning training set. MCFA, Medium Chain Fatty Acid.

We have used a dataset of over 200 MAGs from 10 previously published bioreactor experiments to evaluate the prevalence of the emerging microbial ecological model for chain elongation microbiomes. In this model, MCFAs can be produced either from intermediates, such as lactic acid, or directly from carbohydrates. Using machine learning and protein homology predictions, we find that this ecology model is conserved across various microbial communities from bioreactors fed various carbohydrate rich agroindustrial residues. While the MAGs assembled from each microbial community were not found to be identical in terms of sequence similarity, the biological functions of the microbial communities are predicted to be maintained in MAGs from various taxonomic groups with different relative abundances (Figure 9). Below we discuss observations about the organisms classified into each group.

### 4.1 A taxonomically diverse set of MAGs is predicted to ferment carbohydrates to intermediates

The Ferment to Intermediates functional group was comprised of many MAGs classified in the phylum *Firmicutes*, specifically lactic acid bacteria, which are associated with carbohydrate fermentation to lactic acid and other intermediates (Garde et al., 2002; Ganzle and Follador, 2012; Gänzle, 2015; Zhang and Vadlani, 2015). Indeed, *Firmicutes,* specifically those in the family *Lactobacillaceae*, make up a large portion of the microbial community in most of the bioreactors analyzed when using cumulative relative genomic abundance as a measure (Figure 9), suggesting MAGs in this phylum may play a key role in fermentation to intermediates across the agroindustrial residues examined. There were other taxonomic groups classified in this group. MAGs from both family *Atopobiaceae* and family *Bifidobacteriaceae* (phylum *Actinobacteriota*) were found to be fairly abundant in a subset of the experiments, specifically Milk Permeate 1 and 2, as well as Cellulosic Ethanol Thin Stillage and Xylose (Figure 9), which supports previous observations of the relationship between these two families (Scarborough et al., 2018a; Carvajal-Arroyo et al., 2019; Walters et al., 2023). Three MAGs in the class *Bacilli* but not part of the *Lactobacillaceae* family as well as three MAGs in the phylum *Proteobacteria* were both categorized as being in this functional group (Figure 9) and were found to be of high abundance in two Starch-EtOH experiments that were conducted at a higher temperature and did not result in accumulation of MCFA chain elongation products (Figure 2; Table 1) (Fortney et al., 2021).

From a metabolic potential perspective, fermentation to intermediates can be accomplished as homolactic fermentation wherein only lactic acid is produced, or heterolactic fermentation, either by the phosphoketolase pathway or the bifid shunt pathway, wherein lactic acid and other products (ethanol or acetate) are produced (Pokusaeva et al., 2011; Gänzle, 2015). The percentage of detected gene homologs that encode enzymes unique to each fermentative pathway can be used to evaluate which fermentative pathways may be present in each MAG (Supplementary Figure S1A). In the majority of MAGs, greater than 60% of the unique proteins in the homolactic and the heterolactic bifid shunt pathways were detected, suggesting these are the primary sources of lactic acid across the microbial communities. This included the MAGs in the phylum *Proteobacteria* and the non-*Lactobacillaceae* MAGs in the class *Bacilli*, suggesting this is a key reason these MAGs from unexpected taxonomic groups were categorized into this functional group (Supplementary Figure S1A). No MAGs contained more than 60% of the unique proteins in the heterolactic phosphoketolase fermentation pathway, with the majority containing less than 40% of the unique enzymes (Supplementary Figure S1A), suggesting this is a not a key pathway in abundant members of the communities that are found when using these agroindustrial residues. Nearly all the MAGs in the family *Bifidobacteriaceae* have over 80% of the unique enzymes in the heterolactic bifid shunt fermentative pathway, which is to be expected for members of this family (Supplementary Figure S1A) (Pokusaeva et al., 2011). Future research can explore the proposal that these MAGs that perform lactic acid fermentation and do so using the homolactic fermentation pathway or heterolactic bifid shunt fermentation pathway.

### 4.2 MAGs from several taxonomic groups are predicted to use intermediates for chain elongation

The Intermediate Chain Elongators functional group was comprised of MAGs from a variety of taxonomic classifications (Figure 7). While nearly all the MAGs were part of the phyla *Firmicutes_A* or *Firmicutes_C*, the lower taxonomic levels were more differentiated (Figures 7, 9), suggesting a variety of microorganisms capable of performing this transformation in these microbial communities. Several of these MAGs belonged to families included in the training set, supporting the functional classification—*Anaerovoracaceae, Clostridiaceae, Eubacteriaceae,* and *Megasphaeraceae*—and were the MAGs with the highest relative level of genomic abundance in the experimental microbial communities (Figure 9). This suggests that these MAGs may play a key role in converting intermediates to MCFAs. Interestingly, the machine learning approach predicted MAGs from other families may also perform this biological function. These included MAGs from the phylum *Bacteroidia* and the families *Acidaminococcaceae, Lachnospiraceae,* and *Oscillospiraceae* (Figure 7). A member of the family *Oscillospiraceae*, *Caproicibacterium lactatifermentans*, was shown to utilize lactic acid, a function unique from other members of this family (Wang et al., 2022), and the *Oscillospiraceae* MAG has homologs of the key proteins for conversion of lactic acid to MCFAs (Supplementary Figure S1B). MAGs that belong to family *Lachnospiraceae* have been shown to convert carbohydrates directly to MCFAs (Scarborough et al., 2018a; Scarborough et al., 2020), but our analysis suggests they may also convert fermentation intermediates into these products. Indeed, UW_MP_LCO2_1 contains all three proteins key for conversion of lactic acid to MCFAs, supporting a possible alternative role of the MAG from this family (Supplementary Figure S1B).

However, neither the *Lachnospiraceae* MAG nor the *Oscillospiraceae* MAG were highly abundant in any of the datasets analyzed (Figure 9), suggesting they may not play a large role, even if they do generate MCFAs from intermediates. Interestingly, the *Acidaminococcaceae* and *Bacteroidia* MAGs have relatively high abundance in the Milk Permeate 1 experiment (Figure 9), raising the possibility that the unique conditions of that experiment (Walters et al., 2023) may lead to the enrichment of these MAGs to convert fermentation intermediates to MCFAs. However, the two MAGs belonging to phylum *Bacteroidota* are the only two MAGs for which a majority of genes encoding for lactic acid utilization and reverse ß-oxidation were not detected (Supplementary Figure S1B). This raises the possibility that these MAGs were misclassified, but their metabolic potential deserves future exploration since phylogenetically related organisms have recently been associated with SCFA production in microbial communities (Watanabe et al., 2021; Ho et al., 2021; Liu et al., 2022).

### 4.3 MAGs from various taxonomic groups are predicted to use carbohydrates for chain elongation

A majority of the MAGs classified in the Carbohydrate Chain Elongators group by the machine learning algorithm we used belong to the phylum *Firmicutes* and specifically five families: *Lachnospiraceae, Acutalibacteraceae, Bacillaceae, Sporolactobacillaceae,* and *Clostridiaceae* (Figure 9)*.* Of these MAGs, *Lachnospiraceae* has been shown to produce MCFAs from carbohydrates in other microbial communities (Scarborough et al., 2018a; Scarborough et al., 2020). Indeed, the *Lachnospiraceae* MAGs are the most abundant across the largest number of reactor experiments, suggesting they are key players in MCFA synthesis from carbohydrate (Figure 9). Interestingly, for two of these MAGs we were not able to identify homologs to three of the four enzymes involved in chain elongation (Supplementary Figure S1C). While this may indicate mis-classification, it also raises the possibility that other enzymes may perform these processes in these organisms or that the enzymes have diverged enough in these MAGs so the homologs were below our thresholds. Additional research into these MAGs will be required to examine these hypotheses.

Most of the MAGs in this group contain homologs for the chain elongation genes, although many of them outside the *Lachnospiraceae* family also contain at least one homolog of the lactic acid utilization genes (Supplementary Figure S1C). These results suggest that these MAGs may be able to convert both carbohydrates as well as lactic acid into MCFAs. This has been observed in other microbes including *Caproicibacterium lactatifermentans* (family *Acutalibacteraceae*) (Wang et al., 2022) and *Megasphaera hexanoica* (family *Megasphaeraceae*) (Jeon et al., 2017; Kang et al., 2022). Interestingly, MAGs within the same family (*Acutalibacteraceae*) differ in the presence of lactic acid utilization homologs (Supplementary Figure S1C), suggesting this difference may be on the genus or species level. Recent results suggest members of this family can produce MCFAs from lactic acid (Wang et al., 2022) as well as carbohydrates (Van Nguyen et al., 2023). Further research into these MAGs and related isolated organisms will be valuable to evaluate this new hypothesis.

Of the two MAGs in the class *Bacilli* that are classified as Carbohydrate Chain Elongators, UW_MP_SPOR1_1 (family *Sporolactobacillaceae*) lacked homologs to the electron bifurcating acyl-CoA dehydrogenase and the acetyl-CoA C-acetyltransfase enzymes while UW_TS_BAC2_1 (family *Bacillaceae*) contained homologs for all examined enzymes (Supplementary Figure S1C). Members of the family *Sporolactobacillaceae* are known to produce lactic acid (Chang et al., 2008; Tolieng et al., 2017), so our findings raise the possibility that some members of class *Bacilli* may be able to produce MCFAs as well. Similarly, the MAG in the family *Clostridiaceae* contained homologs for all enzymes examined, including the lactic acid utilization proteins, suggesting that this MAG may produce MCFAs from lactic acid as well as carbohydrates. Members of the phyla *Spirochaetota* and *Proteobacteria* are not known to perform chain elongation, but the MAGs contain at least some of the genes encoding enzymes important for chain elongation, raising the possibility of an expanded functional role of MAGs from these taxonomic groups (Supplementary Figure S1C). Taken together, the results from the machine learning analysis both support previous research and suggest potential new groups of organisms that may be able to perform the specific biological function.

### 4.4 Phylogenetic analysis of EtfB homologs can differentiate between lactic acid utilization and chain elongation

**FIGURE 10:**
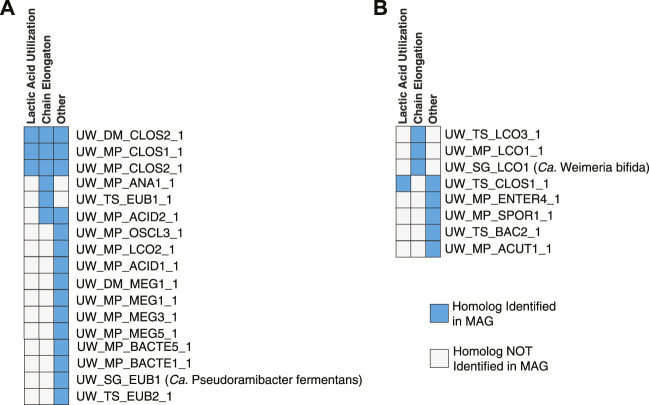
Association of EtfB homologs with lactic acid utilization, chain elongation, or other functions. Summary of the phylogenetic analysis (Supplementary Figure S2) examining EtfB homologs in the MAGs from the Intermediate Chain Elongators group **(A)** and the Carbohydrate Chain Elongators group **(B)**. MAGs with an EtfB homolog that the phylogenetic analysis suggests is associated with lactic acid utilization have a blue box in the first column while MAGs with an EtfB homolog that the phylogenetic analysis suggests is associated with chain elongation have a blue box in the second column. A blue box in the Other column indicates that a MAG has an EtfB homolog for which the phylogenetic analysis cannot indicate a clear function.

The electron flavoprotein (EtfAB) can form a complex with both electron confurcating lactate dehydrogenase (ecLDH, involved in lactic acid utilization) and acyl-CoA dehydrogenase (ACD, involved in chain elongation) (Garcia Costas et al., 2017; Detman et al., 2019) and phylogenetic analysis of the beta subunit (EtfB) can be used to differentiate between the ability to use lactic acid and to perform chain elongation (Walters et al., 2023). This analysis suggests that three MAGs in the Intermediate Chain Elongators group contain multiple copies of EtfB, one associated with ecLDH and one associated with ACD (Figure 10; Supplementary Figure S2), supporting the functional classification that these MAGs use lactic acid to perform chain elongation. Three MAGs in the Carbohydrate Chain Elongators group contain a single copy of EtfB associated with ACD (Figure 10; Supplementary Figure S2), supporting the classification that these MAGs can produce MCFAs but not utilize lactic acid. However, a majority of the MAGs in both functional groups contain EtfB homologs for which the phylogenetic analysis cannot predict a metabolic function. Additional research into the metabolism of microorganisms represented by these MAGs will be required to elucidate the function of these EtfB homologs.

### 4.5 Additional data needed to better understand and predict operation of these microbial communities

All of the analyses in this study were performed using metagenomic data for the MAGs across the 10 experiments. Importantly, metagenomics data can inform what genes are present in a microbial community, and thus we can use this presence to classify MAGs using machine learning. However, presence of a gene does not indicate how much that gene is expressed and thus how important the protein is to the microbial community. Previous work has shown a dramatic disconnect in MAG abundance when calculated using metagenomics (DNA) data or metatranscriptomics (RNA) data (Jewell et al., 2016; Lawson et al., 2017; Beach et al., 2021; Watanabe et al., 2021). The addition of metatranscriptomics to study this ecological microbial model would not only indicate the expression level of the genes in each MAG, but would also provide more information about the functional abundance of each MAG within each functional group.

For the machine learning analysis, we selected isolated bacteria that had been shown to perform the biological function for each group. This meant we were limited in how many organisms were available to use to build our training set. One key example is the lack of isolated organisms shown to convert ethanol to MCFAs. The only isolated organism we were able to find supported evidence for this biological process was the well-studied species *Clostridium kluyveri* (Seedorf et al., 2008; Han et al., 2018). Due to the limited available genomes that represent isolated organisms known to produce MCFA from ethanol by chain elongation, we did not attempt to predict this as a separate functional group. As more bacteria are isolated and studied for this biological process, it is likely the machine learning model can be updated to distinguish between MAGs that using ethanol and those that use lactic acid to produce MCFAs, adding more value to this type of classification procedure.

This study suggests that the ecological microbial model of different functional groups (Ferment to Intermediates, Intermediate Chain Elongators, and Carbohydrate Chain Elongators) is common among microbial communities enriched in carbohydrate-rich agroindustrial residues seeded with anaerobic digester sludge from the wastewater treatment plant. Examination of a microbial community enriched in food waste, a carbohydrate-rich liquid medium, and an inoculum of anaerobic digester sludge from a wastewater treatment plant suggested a similar ecological model (Crognale et al., 2021). A key question that remains is how widespread this ecological model is when applied to other microbial communities, especially in terms of different inocula and feedstock used. Additional research into the composition and genomic make up of other microbial communities would be fascinating and reveal how universal this model is among microbial communities performing chain elongation to produce MCFAs.

### 4.6 Concluding remarks

Examining the 240 MAGs across 10 experiments provided us an opportunity to develop new tools to better understand the microbial communities present across the bioreactors. Specifically, the large data set enabled the use of multiclass classification machine learning to categorize the MAGs into distinct functional groups in an unbiased manner. These tools can be adapted to evaluate other microbial ecology models by changing or expanding the functional groups included in the models. Thus, this analysis not only further explained the core functional groups for MCFA production in carbohydrate rich agroindustrial residues but also demonstrated a new way to quickly examine and explore microbial communities. Such knowledge will help generate hypotheses about microbial community members that could be experimentally tested, helping in the development of better strategies to manage microbiomes to produce desired products, as well as to better characterize microbial functions in a wide variety of microbiomes.

## Data Availability

The datasets presented in this study can be found in online repositories. The names of the repository/repositories and accession number(s) can be found below: https://www.ncbi.nlm.nih.gov/, PRJNA768492, https://www.ncbi.nlm.nih.gov/, PRJNA418244, https://www.ncbi.nlm.nih.gov/, PRJNA535528, https://www.ncbi.nlm.nih.gov/, PRJNA518398, https://www.ncbi.nlm.nih.gov/, PRJNA518399, https://www.ncbi.nlm.nih.gov/, PRJNA518400.
