# Supplementary material for: Comparison of metagenomes from fermentation of various agroindustrial residues suggests a common model of community organization
Source: Front Bioeng Biotechnol. 2023 May 10;11:1197175. doi: 10.3389/fbioe.2023.1197175 (PMC10228549; doi:10.3389/fbioe.2023.1197175)
Supplement: Supplementary file 9 [file DataSheet1.docx]

Supplementary Material

Comparison of metagenomes from fermentation of various agroindustrial residues suggests a common model of community organization

**Kevin S. Myers^1,2^; Abel T. Ingle^1,2,3^; Kevin A. Walters^1,2,3^; Nathaniel W. Fortney^1,2‡^; Matthew J. Scarborough^4^, Timothy J. Donohue^1,2,5^, Daniel R. Noguera*^1,2,3^**

^1^ Great Lakes Bioenergy Research Center, University of Wisconsin-Madison, Madison, WI, USA

^2^ Wisconsin Energy Institute, University of Wisconsin-Madison, Madison, WI, USA

^3^ Department of Civil and Environmental Engineering, University of Wisconsin-Madison, Madison, WI, USA

^4^ Department of Civil and Environmental Engineering, University of Vermont, Burlington, VT, USA

^5^ Department of Bacteriology, University of Wisconsin-Madison, Madison, WI, USA

**^‡^**Current affiliation: Thermo Fisher Scientific, Middleton, WI, USA

*** Correspondence:**Daniel R. Noguera
dnoguera@wisc.edu

# Supplementary Tables

**Table S1.** 240 MAGs collected from the inoculum and 10 experiments with different agroindustrial residues. (Excel File)

**Table S2.** 217 MAGs deemed non-redundant by dRep. (Excel File) **Table S3.** Organization and distribution of GTDB-Tk determined taxonomy for all 217 non-redundant MAGs. (Excel File)
 **Table S4.** 131 MAGs with at least 1% relative abundance in at least 1 experimental sample and machine learning functional group classification. (Excel File)
 **Table S5.** Enzyme list and reaction presence or absence in all MAGs used for metabolic make up of MAGs and genomes. (Excel File) **Table S6.** Genomes and results used as a training set for the machine learning algorithm. (Excel File)

# Supplementary Figures Captions

**Figure S1. Summary of key metabolic pathway and enzyme presence and absence across the MAGs in each functional group.** A) Summary of enzyme presence or absence in three fermentative pathways for each MAG classified in the Ferment to Intermediates group, bold name indicates MAG generated from long-read PacBio sequencing data. The percentage of each protein unique to one of the three fermentative pathways examined (homolactic, heterolactic phosphoketolase, and heterolactic bifid shunt) is represented by the different colored boxes. Note the high abundance of enzymes in the heterolactic bifid shunt for MAGs in the family *Bifidobacteriaceae*. Also shown is the taxonomic grouping from Figure 5. *Pro., Proteobacteria.* B) Summary of enzyme presence or absence known to be required for lactic acid conversion to MCFA for MAGs classified in the Intermediate Chain Elongation group, bold name indicates MAG generated from long-read PacBio sequencing data. Blue boxes indicate protein presence while yellow boxes indicate protein absence for each MAG. Shown is the taxonomic grouping from Figure 6. *Ba.*, *Bacteroidota*; *Euba., Eubacteriales*; *Eub., Eubacteriaceae; Pep., Peptostretococcales; Ana., Anaerovoracaceae; Clostr., Clostridales; Clostrid., Clostridiaceae; Acida., Acidaminococcales; Acida., Acidaminococcaceae; Ls., Lachnospirales; La., Lachnospiraceae; Ol., Oscillospirales; Os. Oscillospiraceae*. C) Summary of enzyme presence or absence known to be required for lactic acid conversion to MCFA for MAGs classified in the Carbohydrate Chain Elongators group, bold name indicates MAG generated from long-read PacBio sequencing data. Shown is the taxonomic grouping from Figure 7. *Pro., Proteobacteria; Osc., Oscillospirales; Acu., Acutalibacteraceae; Baci., Bacillales; So., Sporolactobacillaceae; Bl., Bacillaceae; Cl., Clostridiales; Co., Clostridiaceae; Sp., Spirochaetota.*

**Figure S2. Phylogenetic analysis of EtfB homologs from genomes and MAGs in the Intermediate Chain Elongators and Carbohydrate Chain Elongators functional groups.** A maximum-likelihood phylogenetic tree constructed using RAxML-ng (Kozlov et al., 2019) with 500 bootstraps (values >50 shown) of the EtfB homologs identified from genomes previously used (black genome name) (Walters et al., 2023), MAGs in the Intermediate Chain Elongators group (orange MAG name), and the Carbohydrate Chain Elongators group (green MAG name). More than one EtfB homolog could be identified in each MAG and is indicated with numbers. The scale bar indicates the number of nucleotide substitutions per sequence site. Genomic position was determined previously (Walters et al., 2023). Genomes wherein *etfB* was in the same neighborhood as lactic acid utilization genes encoding electron confurcating lactate dehydrogenase (ecLDC) and lactate permease (LacT) are indicated with green circles. Genomes wherein *etfB* was in the same neighborhood as chain elongation genes encoding acyl-CoA dehydrogenase (ACD), acetyl-CoA acetotransferase (ACAT), 3-hydroxyacyl-CoA dehydrogenase (HAD), and enoyl-CoA hydratase (EcOAH) are indicated with purple circles. Green bars indicate MAGs and genomes where in EtfB is predicted to associate with lactic acid utilization proteins and purple bars indicate MAGs and genomes where EtfB is predicted to associate with chain elongation proteins.
